# Biosynthesis of gold nanoparticles in the fruiting body of enoki mushrooms (*Flammulina velutipes*) under Pb^2+^ induction

**DOI:** 10.1049/nbt2.12104

**Published:** 2022-11-19

**Authors:** Jingang Mo, Jun Jin, Han Yu, Mingjun Ai, Die Hu, Linlin Li, Kai Song

**Affiliations:** ^1^ School of Life Science Changchun Normal University Changchun China; ^2^ Institute of Science, Technology and Innovation Changchun Normal University Changchun China

## Abstract

Fungi can produce many compounds, such as proteins, enzymes, amino acids, and polysaccharides, which are internalised and enriched for metals, and are widely used as reducing and stabilising agents for the biosynthesis of gold nanoparticles (Au NPs). Almost all fungal sources used in the synthesis of the Au NPs are in the form of cell filtrates or mycelial suspensions. However, the culture of cell‐free fungal filtrate and mycelium is not comparable to the propagation of fungal substrates in input and operation. Here, we evaluated in vivo biosynthesis of Au NPs in enoki mushrooms (*Flammulina velutipes*). HAuCl_4_ was reduced in the fruiting body of the enoki mushrooms via induction by Pb^2+^, resulting in the generation of Au NPs. We then employed UV‐Vis absorption spectroscopy, Transmission Electron Microscope, and Energy Dispersive Spectrometer to characterise various shapes of the Au NPs. The elemental analysis indicated that the Au NPs were mainly concentrated in organelles of the stalk and cap cells. We also demonstrated that 0.3–0.5 mM HAuCl_4_ was the optimal stress treatment concentration based on the changes in physiological indicators of the enoki mushrooms. This work reveals that fungi can be utilised well as nanomaterial bioreactors.

## Graphical abstract

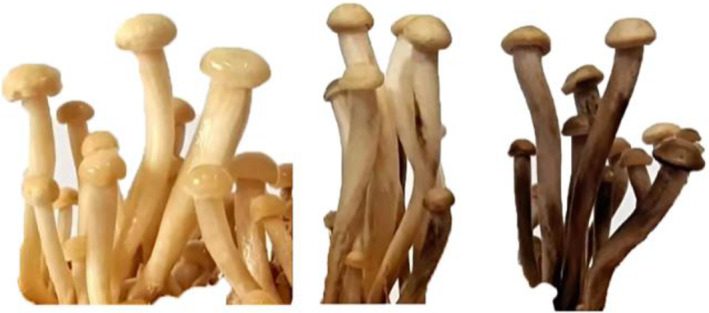

HAuCl4 was reduced by fruiting body of the enoki mushrooms via induction by Pb2+, which resulted in production of Au NPs.

## 1 INTRODUCTION

Au NPs are frequently used in the domains of optics, electronics, and catalysis because of their distinct physical and chemical characteristics. The current standard physical and chemical synthesis methods are associated with high energy consumption, intense pollution, and slow speed [1]. As a result, there is a lot of interest in the development of green and sustainable synthesis processes. Due to their high tolerance and prolific enzyme production, microbial organisms like fungus have been the subject of several studies evaluating their biomineralisation mechanisms [2, 3, 4].

The synthesis of fungal nanomaterials is achieved because fungi can metabolically secrete many active substances, such as extracellular proteins, and that the synthesised nanomaterials can easily be separated from the organism. Previous researchers have reported the synthesis of Au NPs using *Verticillium* *sp*., *Colletotrichum* *sp*., *Aspergillus niger*, *Helminthosporium solani*, *Trichoderma koningii*, *Rhizopus stolonifer*, *Penicillium* sp., *Epicoccum nigrum*, *Neurospora crassa and Cylindrocladium floridanum*. The Au NPs are of various shapes, including spherical, rod‐shaped, triangular, pentagonal, star‐shaped regular nanoplates, helical nanoplates, layered nanoclusters agglomerates and spherical nano‐agglomerates, and range between 1 and 50 nm [5, 6, 7, 8, 9, 10, 11, 12, 13, 14, 15, 16].

Almost all fungal resources synthesising Au NPs are in the form of cell filtrates or mycelial suspensions [17, 18]. Although it is theoretically simple to control the synthesis of extracellular Au NPs, the input and operation of the growth of cell‐free fungal filtrate and mycelium are not comparable to those of the propagation of fungal substrates. Inspired by the previous work on mycosynthesis of Au NPs using the extract of *Flammulina velutipes* [19], we exploited the super heavy metal enrichment ability of the fruiting body of enoki mushrooms to allow direct reduction of HAuCl_4_ for in vivo biosynthesis of Au NPs. In addition, we explored the optimised conditions under Pb^2+^ induction, characterised the Au NPs particle size, morphology, and surface state, and investigated changes in physiological indicators of enoki mushrooms. The yield of Au NPs obtained from enoki mushrooms has not yet achieved the acceptable level; however, in terms of the properties of as‐prepared Au NPs, it can be employed in a manner quite similar to the method of obtaining Au NPs using fungal extracts.

## 2 MATERIALS AND METHODS

### 2.1 Optimisation of growth conditions of the fruiting body of enoki mushrooms

Enoki mushroom roots were cleaned three times with distilled water before being blotted on filter paper. A petri dish with a diameter of 10 cm was filled with two filter sheets, and roots weighing 25 g and 2 cm in length were planted on the filter papers. The roots were wrapped in plastic and incubated for 15 days at various temperatures: 15, 20, 25, and 30℃. Periodically, the substrate root's length was measured.

### 2.2 Biosynthesis of Au NPs

The fungi culture is carried out in a culture room with fungal culture shelves, temperature control devices, and ventilation equipment. The culture temperature was 25℃ in the early stages to enhance the germination of the mycelium, 20℃ in the middle, and 15℃ in the late stages. The mycelium was rejuvenated to maturation. Culture humidity was kept at 60%, with a CO_2_ concentration of 3000–4000 ml/L, which is optimal for mycelium growth.

After the mycelium grew to the full size of the vial, 7–8 mm of the ageing mycelium was removed and promptly replenished with water. The culture temperature was kept at 15℃, with a relative humidity of 90%–95%, and a CO_2_ concentration of 2000–3000 ml/L. The vials were sprayed with water twice a day and placed under 100% light after 3 days. When the mycelium grew to the formative stage, they underwent treatment with HAuCl_4_ solution (0.01, 0.05, 0.1, 0.3, 0.5, 1 mM HAuCl_4_ solution) with or without 0.1 mmol/L Pb^2+^ and then incubated for 10 days.

### 2.3 Extraction of Au NPs

The Enoki mushroom substrates grown and enriched with Au NPs were placed in a mortar. Liquid nitrogen and Phosphate Buffered Saline buffer were added, and then the substrates were fully ground. The ground homogenate was centrifuged at 8000 rpm for 20 min, and then the supernatant was placed in a refrigerator (4℃) for analysis.

## 3 RESULTS AND DISCUSSION

### 3.1 Analysis of growth conditions for enoki mushrooms

Although it has been shown that the temperature for the formation of enoki mushrooms was 5–19℃, with an optimum temperature of 13–14℃ [20], the growth temperature of enoki mushroom substrates also varies on the species. The results of the pre‐experiments showed that the enoki mushroom's growth hardly continued in conditions above 25℃ (Figures S1 and S2). The higher fresh weight of golden mushroom was obtained at 20℃ and 15℃ respectively. Eventually, the 15℃ culture condition was selected as a more suitable temperature for the growth of the enoki mushrooms.

### 3.2 UV‐vis spectral characterisation

The HAuCl_4_ concentrations we used were optimised and it was found that from the colour and absorption spectra of the enoki mushrooms extracts (Figures S3 and S4), the 0.3 and 0.5 mM HAuCl_4_ treatment groups were preferable, so what follows focuses on the former and the latter as a reference.

**FIGURE 1:**
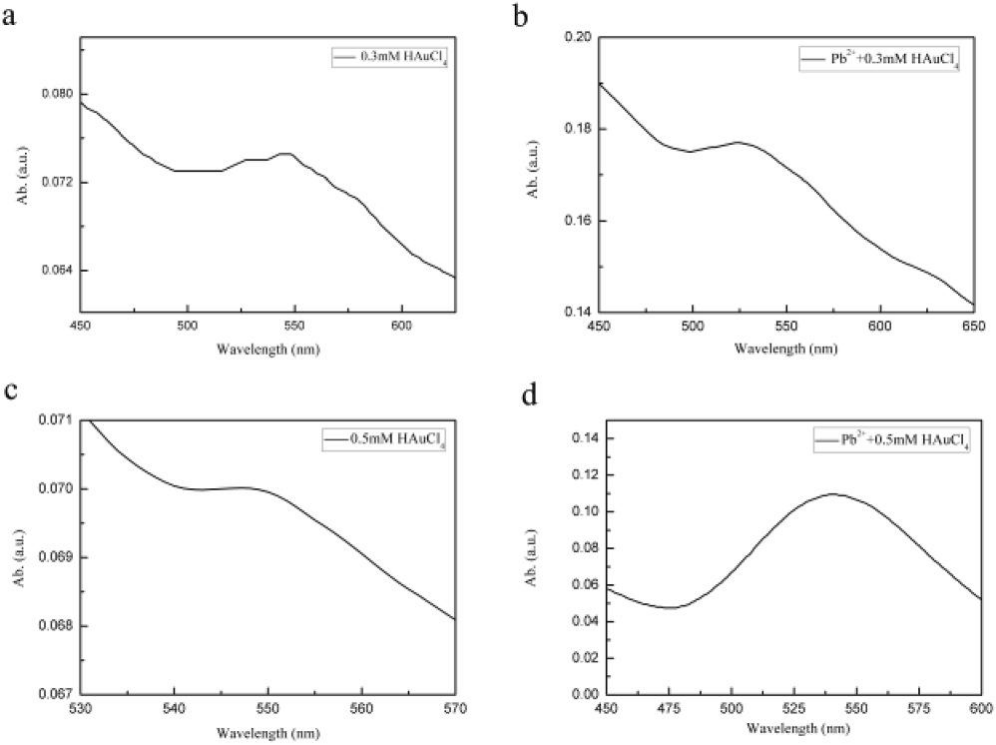
The UV‐vis of gold nanoparticles (Au NPs) synthesised under (a, c) 0.3 and 0.5 mM HAuCl_4_, (b, d) Pb^2+^ induced.

0.3 and 0.5 mM HAuCl_4_, with or without Pb^2+^ induction, had characteristic absorption peaks around 550 nm, with the synthesised Au NPs having subspherical irregular shapes, and other non‐spherical shapes based on the peaks (Figure 1) [21]. A range of concentrations of the HAuCl_4_ solution is reducible to synthesise Au NPs, as shown in Table 1.

**TABLE 1:** UV‐vis characteristic absorption peaks of gold nanoparticles (Au NPs) synthesised under different conditions

Synthetic conditions		UV‐visible characteristic absorption peak (nm)
HAuCl_4_ concentration (mM)	With (+) or without (−) Pb^2+^ induction	
0.01	−	548
	+	544
0.05	−	550
	+	545
0.1	−	552
	+	545
0.3	−	550
	+	542
0.5	−	550
	+	542
1	−	550
	+	542

### 3.3 TEM characterisation

**FIGURE 2:**
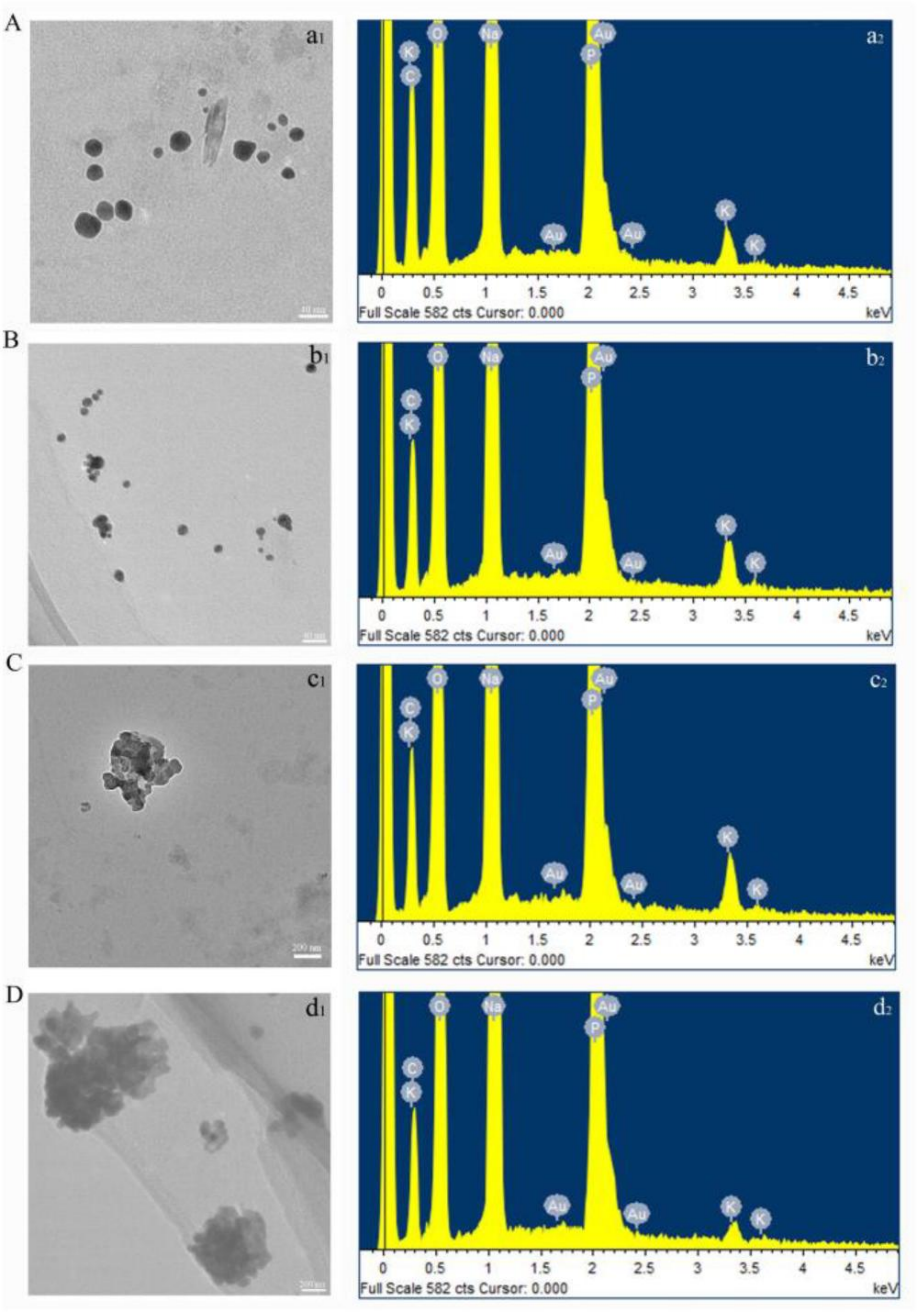
The Transmission Electron Microscope and Energy Dispersive Spectrometer of the gold nanoparticles (Au NPs) synthesised under (a) 0.3 mM HAuCl_4_, (b) 0.3 mM HAuCl_4_ induced by Pb^2+^, (c) 0.5 mM HAuCl_4_, (d) 0.5 mM HAuCl_4_ induced by Pb^2+^.

As shown in Figure 2a, near‐spherical and irregularly shaped Au NPs were synthesised under 0.3 mM HAuCl_4_, and the Energy Dispersive Spectrometer further demonstrated the formation of Au NPs. The results showed that their agglomeration was apparent, which was in sync with the UV‐vis spectra. The maximum particle size was 115.386 nm, an aggregate of Au NPs, while the minimum particle size was 17.871 nm and the average particle size was 50.43 nm. Figure 2b,d shows that Pb^2+^ synthesised subspherical, hexagonal Au NPs and large aggregates under 0.3 and 0.5 mM HAuCl_4_. In addition, Pb^2+^ regulated the formation and secretion of extracellular polymers (glyoxalate and nitrate reductase), which affected the assembly and growth process of the Au NPs, further affecting the particle size of the nanomaterials [22]. The morphology and particle size of the produced Au NPs are shown in Table 2.

**TABLE 2:** Morphology and particle aggregates size of the gold nanoparticles (Au NPs) synthesised under different conditions

Synthetic conditions		Characteristics of Au NPs			
HAuCl_4_ concentration (mM)	With (+) or without (−) Pb^2+^ induction	Maximum particle size (nm)	Minimum particle size (nm)	Average particle size (nm)	Morphology
0.3	−	115.386	17.871	50.43	Irregularly shaped, subspherical
	+	536.122	11.616	321.516	Subspherical, hexagonal, quadrilateral
0.5	−	71.775	12.346	38.884	Irregularly shaped, subspherical
	+	154.087	3.659	41.323	Irregularly shaped, subspherical

### 3.4 Zeta potential and FTIR characterisation

Due to the limitations of the purification process and reaction conditions, we have not yet been able to obtain Au NPs with excellent dispersion. Therefore, such a large value of positive charge for Zeta potential results is most likely due to the surface charge of the Au NPs aggregates (Table 3). Although we do not have access to the surface charge of individual Au NPs at present, it can be assumed from the extent of Au NPs aggregates in Transmission Electron Microscope that the exact value of the surface charge should be much less than +30 mV.

**TABLE 3:** Zeta potential of gold nanoparticles aggregates under different conditions

HAuCl_4_ concentration (mM)	With (+) or without (−) Pb^2+^ induction	Zeta potential (mV)
0.3	−	478.7
	+	639.2
0.5	−	527.2
	+	715.5

**FIGURE 3:**
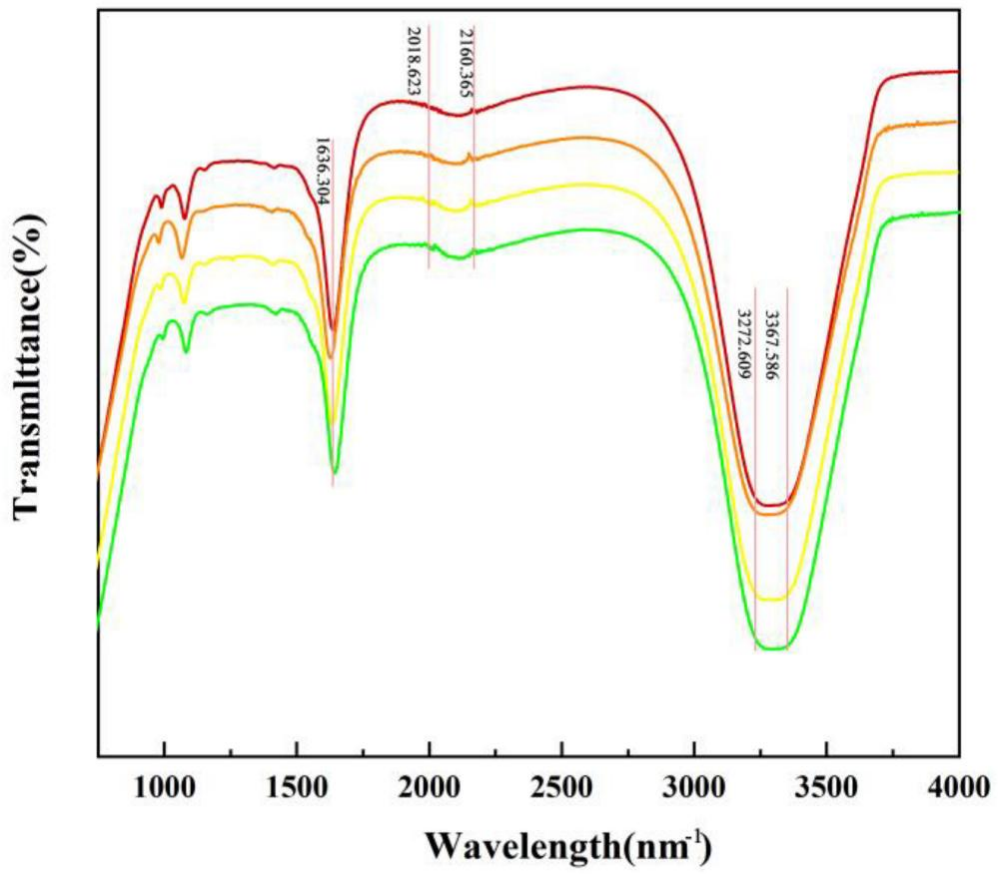
Fourier Transform infrared spectroscopy of the gold nanoparticles (Au NPs) synthesised under different conditions (a) 0.3 mM HAuCl_4_, (b) 0.3mM HAuCl_4_ induced by Pb^2+^, (c) 0.5 mM HAuCl_4_, (d) 0.5mM HAuCl_4_ induced by Pb^2+^.

It was shown that the sugars contained in the enoki mushrooms (glucose, rhamnose, lactose, maltose, fructose, galactose, mannose, xylose, arabinose, and fucose) might be involved in the synthesis of the Au NPs [23]. This was corroborated by the characteristic absorption peak maps of the infrared spectra of the Au NPs. The IR absorption peaks at 1636.304 cm^−1^ were due to the stretching vibration of –C = C–, 2018.623, 2160.365 cm^−1^ were due to the stretching vibration of –C≡C–, while 3272.609, 3367.586 cm^−1^ were due to the stretching vibration of alcohols and phenols with hydroxy‐OH (Figure 3). These data indicated that –C = C–, –C≡C–, and –OH play a role in the synthesis of the Au NPs.

In contrast to the manufacture of plant extracts, the Au NPs so generated are challenging to identify and purify from biological matrices [24], resulting in a weak signal for characterisation of properties. However, there is still a lot of application potential for this in situ synthesis. The body of the enoki mushroom held the produced Au NPs, allowing for easy control of the size and concentration of the metal nanoparticles immobilised on the biological matrix. This was accomplished by physically breaking the fungal body into tiny pieces. These metal nanoparticles are anticipated to serve as catalysts for catalytic reactions, similar to immobilised enzymes. This work indicates the viability of using fungi as nanomaterial bioreactors.

### 3.5 Distribution of Au NPs in the cotyledons of enoki mushrooms

**FIGURE 4:**
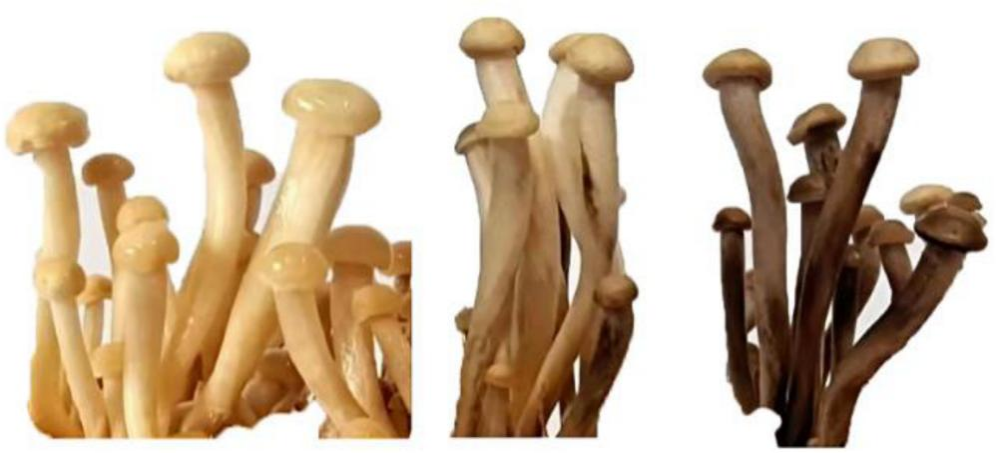
The process of synthesising ‘real gold’ mushrooms.

As seen in Figure 4, the enoki mushrooms' colour gradually turned purple‐black, and the morphological change was not noticeable after 7 days. The substrates were now enriched with Au NPs while still being in a growing state.

**FIGURE 5:**
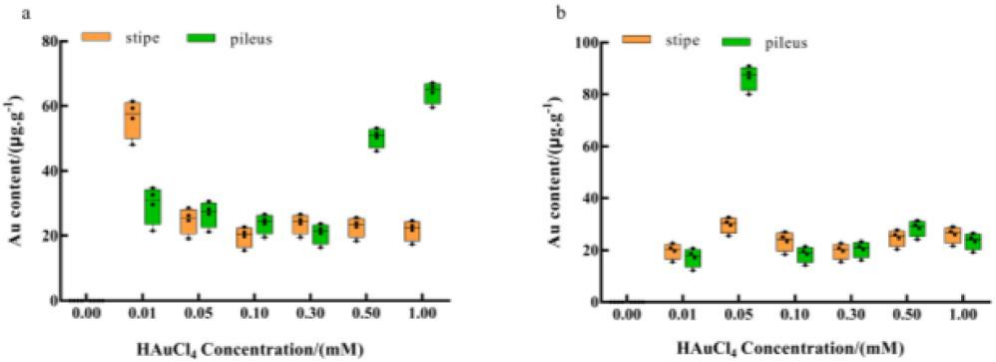
Effect of (a) HAuCl_4_ solution and (b) Pb^2+^ on Au content in different parts of fruiting body. The comparisons within groups are indicated by lowercase letters, with different letters indicating significant differences (*p* < 0.05).

Under Au^3+^ single stress treatment, the Au content in the stalk was greater than that in the cap at low concentrations (0.01 mM). The difference in the Au content was not significant at 0.05–0.3 mM, and the Au content in the cap was significantly greater than that in the stalk at high concentrations (0.5–1 mM). Under Pb^2+^ induction, the difference in the Au content between the stalk and the cap of the ascomata was not significant at all concentrations, but only at 0.05 mM, where the Au content in the cap was significantly higher than that in the stalk (Figure 5).

**FIGURE 6:**
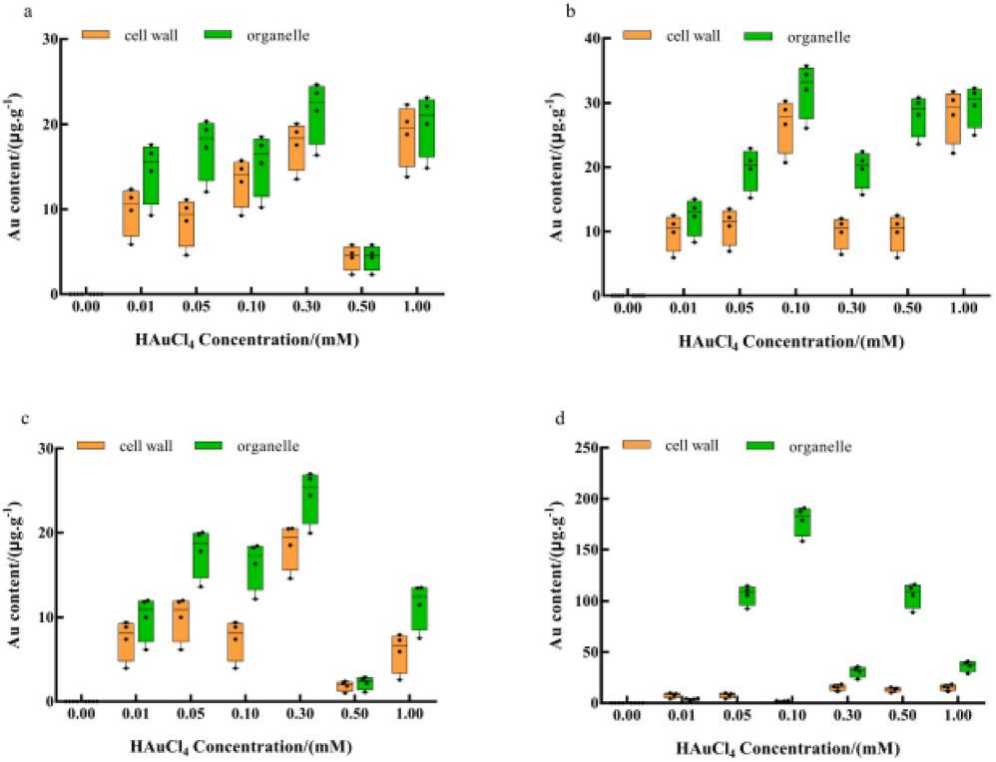
Effects of HAuCl_4_ of (a) stipe and (b) cap and (c) stipe and (d) cap induced by Pb^2+^ on the content of Au in subcells. The comparisons within groups are indicated by lowercase letters, with different letters indicating significant differences (*p* < 0.05).

Previous physiological and biochemical analysis showed that the detoxification mechanism of golden mushroom for Au^3+^ may be associated with converting highly toxic heavy metal ions into less toxic ones through intracellular redox and methylation [25, 26, 27]. Besides, it can be detoxified by metabolising specific proteins, such as metallothioneins, which directly interact with heavy metal ions through regionalisation [28, 29]. Au elements were found in the stalk and cap of the organelle and cell wall after they were subjected to Au^3+^ single stress treatment and Pb^2+^ induction (Figure 6). Similar to how plants detoxify heavy metals, this process uses cellular compartmentalisation [30, 31].

**FIGURE 7:**
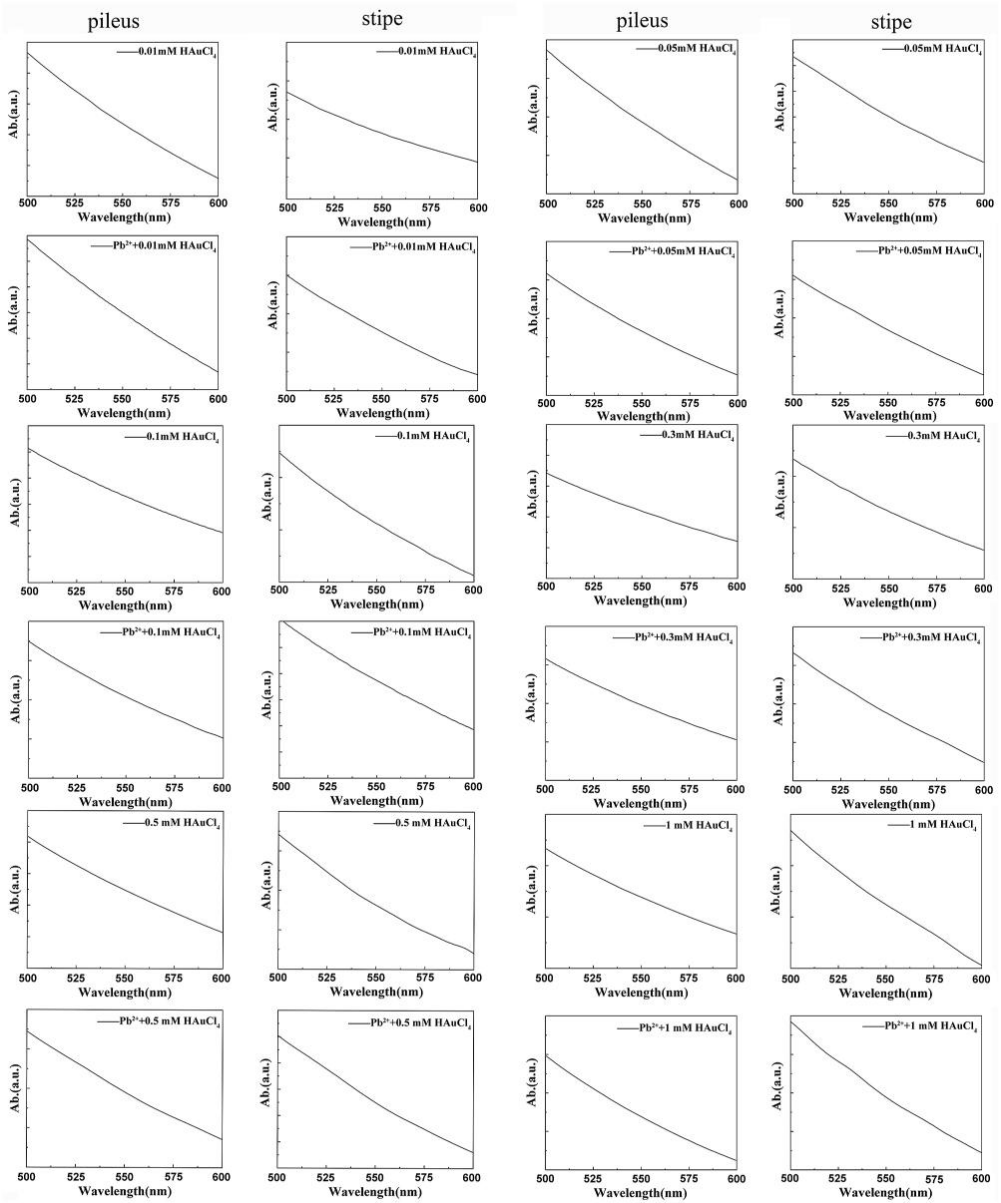
UV‐vis absorption spectra of cytoplasmic extracts of fungal caps and stalks induced by different concentrations of HAuCl_4_ and Pb^2+^.

The stalk's cytoplasm displayed the typical absorption peaks at 550–570 nm at concentrations of 0.05, 0.5, and 1 mM HAuCl_4_. The stalk's cytoplasm displayed the typical absorption peaks at about 530–535 nm under Pb^2+^ induction, showing that small amounts of Au were present. In the cytoplasm of the cap and stalk, the presence or absence of Au NPs is indicated by the absence of a distinctive absorption peak in the range of 600 nm (Figure 7).

### 3.6 Changes in the antioxidant system of the enoki mushrooms

**FIGURE 8:**
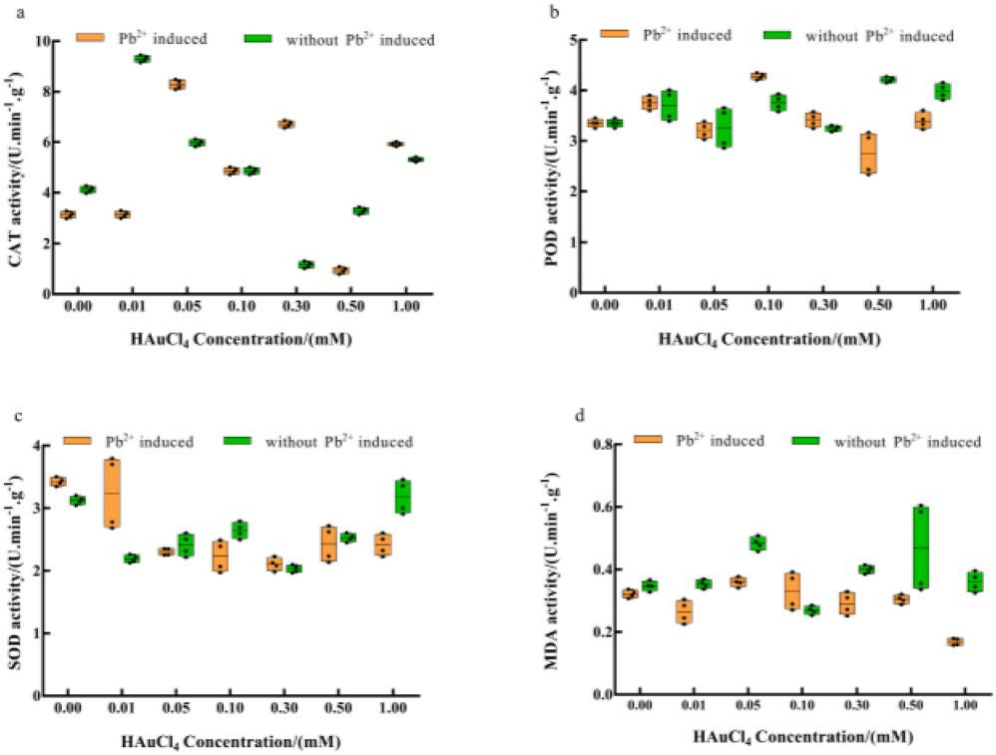
Effect of HAuCl_4_ solution on the antioxidant system (a) catalase (CAT); (b) peroxidase (POD); (c) superoxide dismutase (SOD) and (d) malonic dialdehyde (MDA) content of the enoki mushrooms. The comparisons within groups are indicated by lowercase letters, with different letters indicating significant differences (*p* < 0.05).

Under Au^3+^ single stress treatment, Figure 8 demonstrates a substantial overall pattern of ‘promotion followed by inhibition’ of catalase (CAT) activity, with 0.01 mM producing the maximum promotion and 0.3 mM exerting the maximum inhibition. Peroxidase (POD) activity was generally insignificantly promoted, and the maximum promotion was attained at 0.5 mM. However, Superoxide dismutase (SOD) activity was decreased, with the highest level of inhibition occurring at 0.3 mM. Malonic dialdehyde (MDA) content was generally not promoted, and the greatest promotion was attained at 0.05 mM. Previous research indicated that the HAuCl_4_ solution's foreign stress has an impact on the antioxidant enzyme system's activity, making the organism less able to withstand stress and increasing MDA [32]. The MDA level rises with an increase in Au^3+^ stress concentration, harming the organism. Furthermore, stress from the environment may increase the organism's resilience, reducing the MDA content [33]. It has been demonstrated that Chlorella pyrenoidosa's MDA concentration keeps rising in order to withstand heavy metal Zn^2+^ stress. A drop in the MDA level occurs as a result of cellular damage that occurs after a period of stress, which prevents the organism from continuing to fight the stress [34]. While low doses have little impact, high amounts of heavy metal stress enhance the MDA content in *Halamphora veneta (Kützing) Levkov* [35]. When enoki mushrooms were under heavy metal Au^3+^ stress, this was similar to the overall trend in the MDA level of the mushrooms.

Studies have shown that heavy metal‐induced conditions such as Cd^2+^, Co^2+^, Pb^2+^, Cu^2+^, Hg^2+^, Bi^3+^, Fe^3+^, Mn^2+^, and Zn^2+^ can promote the synthesis of nanomaterials such as Se NPs [36]. For instance, Co^2+^ causes clustering of Au NPs synthesised by *Trichoderma* sp., Pb^2+^ and Sn^2+^ fuel the synthesis rate of *Trichoderma* sp., while Pb^2+^ induces synthesis of homogeneous nanorods with uniform morphology by *Aspergillus* sp. [37, 38]. However, adding Pb^2+^ triggers strong oxidative stress effects, while free radicals trigger thermal excitation effects and redox atmospheres [39]. In this study, under Pb^2+^ induction, there was an overall trend of ‘promotion followed by inhibition’ of CAT activity. The effect on the trend was more significant with increased Au^3+^ concentration, which saw a maximum promotion at 0.05 mM while 0.5 mM exerted maximum inhibition. The effect on POD activity showed a non‐significant ‘promotion followed by inhibition’ trend, and the degree of enhancement reached the maximum at 0.1 mM, while 0.5 mM had the maximum degree of inhibition. In addition, SOD activity was inhibited, and the maximum degree of inhibition was achieved at 0.3 mM. The overall ‘promotion followed by inhibition’ trend for the MDA content was not significant. The maximum promotion and inhibition were achieved at 0.05 and 1 mM respectively.

### 3.7 Effect of osmoregulatory substances in enoki mushrooms

**FIGURE 9:**
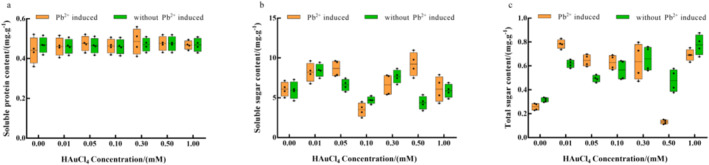
Effect of HAuCl_4_ solution on osmotic substances (a) soluble protein; (b) soluble sugar; (c) total sugar of enoki mushrooms.

Living things and biofilms are protected by osmoregulatory and nutritive components such soluble sugars, soluble proteins, and total sugars. They are crucial to understanding the overall metabolism of the plant body because their contents can predict whether an organism will experience heavy metal stress, drought, or salt resistance under environmental conditions [40]. There was a non‐significant trend towards inhibiting soluble protein content and total soluble sugar content under Au^3+^ single stress treatment settings, with the maximum promotion reported at 0.01 mM and inhibition at 0.1 and 0.5 mM. Additionally, there was a propensity to increase the concentration of total sugar, with the biggest increase occurring at 1 mM (Figure 9).

Under Pb^2+^ induction, there was an overall but non‐significant promotion of soluble protein content and effect of soluble sugar content, with 0.5 mM yielding the most significant promoting effect, and 0.1 mM exerting most significant inhibitory effect. Besides, there was overall enhancement of total sugar concentration, with the highest promoting effect observed at 0.01 mM while the best inhibitory effect was achieved at 0.5 mM. It was presumed that with the changes in the activity of various enzymes in the cells, the rate of metabolism of substances in the organism slows down, leading to their elevated levels [41]. Overall, Pb^2+^ induction did not exert any significant difference on the soluble protein content, soluble sugar content, or total sugar concentration.

### 3.8 Scavenging of DPPH· and ·OH in the enoki mushrooms

**FIGURE 10:**
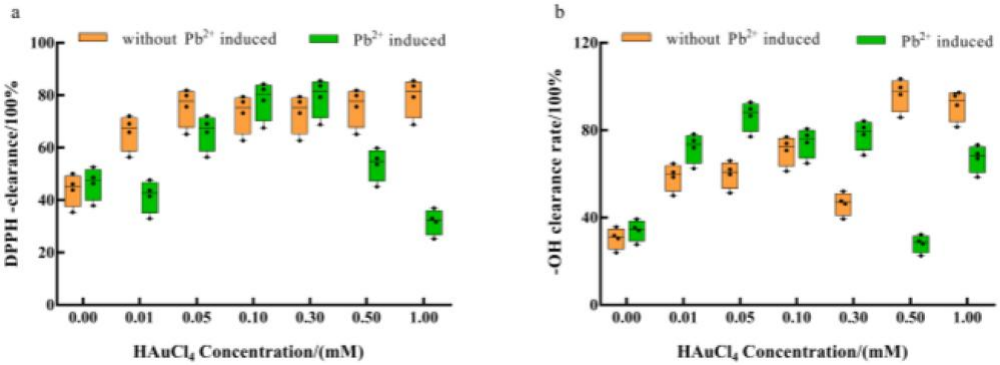
Effect of HAuCl_4_ solution on (a) DPPH· scavenging ability and (b) ·OH scavenging ability of enoki mushrooms. The comparisons within groups are indicated by lowercase letters, with different letters indicating significant differences (*p* < 0.05).

Our results demonstrated that the diphenyl picryl hydrazinyl radical (DPPH·) clearance rate was significantly increased under Au^3+^ single stress treatment. The maximum DPPH· clearance rate (76.39%) was achieved at 1 mM (Figure 10a). Under Pb^2+^ induction, DPPH· clearance showed an ‘increase followed by inhibition’ trend, with 0.3 mM yielding the highest DPPH· clearance (79.68%), while the lowest DPPH· clearance (32.09%) was achieved at 1 mM. Longitudinally, this data demonstrated that Pb^2+^‐induction was associated with higher DPPH‐clearance compared to Au^3+^, at the same concentration (0.1–0.3 mM), while DPPH· clearance was higher for Au^3+^ under other conditions.

The ·OH scavenging rate was significantly higher under Au^3+^ single stress treatment. The ·OH scavenging rate was as high as 97.86% at 0.5 mM. Under Pb^2+^‐induced conditions, the ·OH scavenging rate was significantly higher in all cases, and the highest scavenging rate (86.58%) was achieved at 0.05 mM. Longitudinally, our data showed that at low concentrations (0.01–0.3 mM), Pb^2+^ induced higher ·OH clearance compared to Au^3+^, whereas, at high concentrations (0.5–1 mM), the ·OH clearance was higher for Au^3+^ (Figure 10b). This is related to the substrates' structure and type of antioxidants [42].

### 3.9 Change of active oxygen (O_2_^−^) content in enoki mushrooms

**FIGURE 11:**
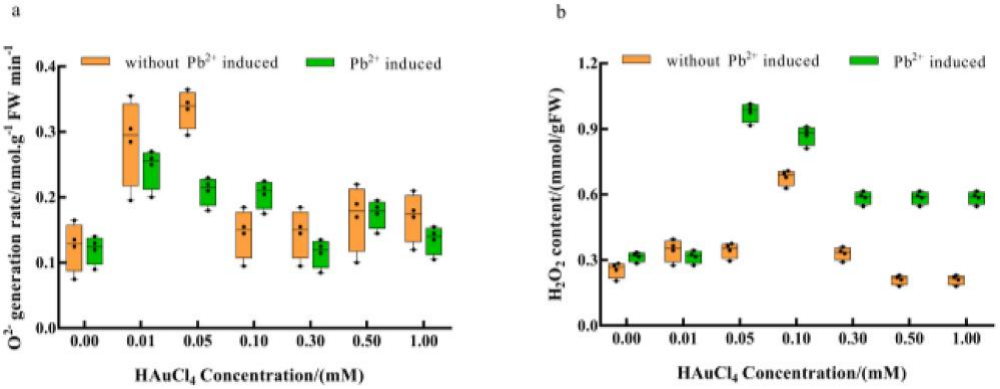
Effects of HAuCl_4_ solution on (a) superoxide anion and (b) hydrogen peroxide content in enoki mushrooms. The comparisons within groups are indicated by lowercase letters, with different letters indicating significant differences (*p* < 0.05).

Au^3+^ single stress treatment exerted an inhibitory effect on O_2_^−^ production rate and a facilitative effect on H_2_O_2_ content. The inhibitory effect on O_2_^−^ production rate reached the maximum at 0.1 mM, while the H_2_O_2_ content reached the maximum at 0.1 mM (Figure 11). Pb^2+^ induction had an inhibitory effect on the O_2_^−^ production rate and a facilitative effect on the H_2_O_2_ content. The inhibitory effect on the O_2_^−^ production rate reached the maximum at 0.3 mM, while the H_2_O_2_ content reached the maximum at 0.05 mM. Longitudinally, our data showed that the O_2_^−^ production rate was higher under Pb^2+^ induction than Au^3+^ at 0.1 mM, while Pb^2+^ induction showed lower performance at other concentrations of Au^3+^. Besides, Pb^2+^ induction led to higher H_2_O_2_ content than Au^3+^ at the same concentration. This outcome was attributed to the disruption of the intracellular membrane structure due to HAuCl_4_ solution stress, which alters the structure and type of antioxidants in the organism, leading to differences in the scavenging capacity of ·OH as well as the H_2_O_2_ and O_2_^−^ content [43].

## 4 CONCLUSION

By reducing Au^3+^ ions in the cotyledon cells of enoki mushrooms, we were able to create inorganic Au NPs in a safe and controlled manner. This process took advantage of the effective metal ion detoxification system that macrofungi have by nature. The Au NPs were reduced using –C=C, –CC, –OH, and other functional groups. The resulting particles were irregular hexagonal, tetragonal, subspherical, or subspherical aggregates with positively charged surfaces. They were primarily present in the stalk and cap of the Enoki mushrooms, with subcellular distribution in the organelles and cell walls but almost absent in the cytoplasm. The data showed an increase in soluble sugar content, total sugar concentration, DPPH· and ·OH scavenging capacity, and reactive oxygen species content, and there was antioxidant enzyme system activity in the enoki mushroom substrates.

## AUTHOR CONTRIBUTIONS

**Jingang Mo**: Writing – original draft. **Jun Jin**: Data curation. **Han Yu**: Data curation. **Mingjun Ai**: Investigation. **Die Hu**: Methodology. **Linlin Li**: Methodology. **Kai Song**: Conceptualisation.

## CONFLICT OF INTEREST

The authors declare no conflict of interest.

## Supporting information

Supporting Information S1

## Data Availability

The data that support the findings of this study are available on request from the corresponding author. The data are not publicly available due to privacy or ethical restrictions.
