# Supplementary material for: Biosynthesis of gold nanoparticles in the fruiting body of enoki mushrooms (Flammulina velutipes) under Pb2+ induction
Source: IET Nanobiotechnol. 2022 Nov 19;17(2):61–8. doi: 10.1049/nbt2.12104 (PMC10116022; doi:10.1049/nbt2.12104)
Supplement: Supplementary file 1 — Supporting Information S1 [file NBT2-17-61-s001.docx]

**Supplementary Files**

**Effect of HAuCl_4_ solution on osmotic substances Enoki mushrooms**

Superoxide dismutase (SOD) by NBT photochemical reduction, catalase (CAT) by UV spectrophotometer, peroxidase (POD) by guaiacol, soluble proteins by Komas Brilliant Blue, soluble sugars by anthrone, malondialdehyde (MDA) by TBA colourimetric method, total sugars by phenol sulphate, reactive oxygen species, DPPH-scavenging capacity and -OH-scavenging capacity by UV spectrophotometer and Au by flame atomic absorption (AAS).

**FTIR characterization**

Functional groups of the synthesized Au NPs were identified by using an FTIR (Perkin Elmer, USA), where the frequency ranges from 4000–500 cm-1.

**Growth conditions for enoki mushrooms**

**
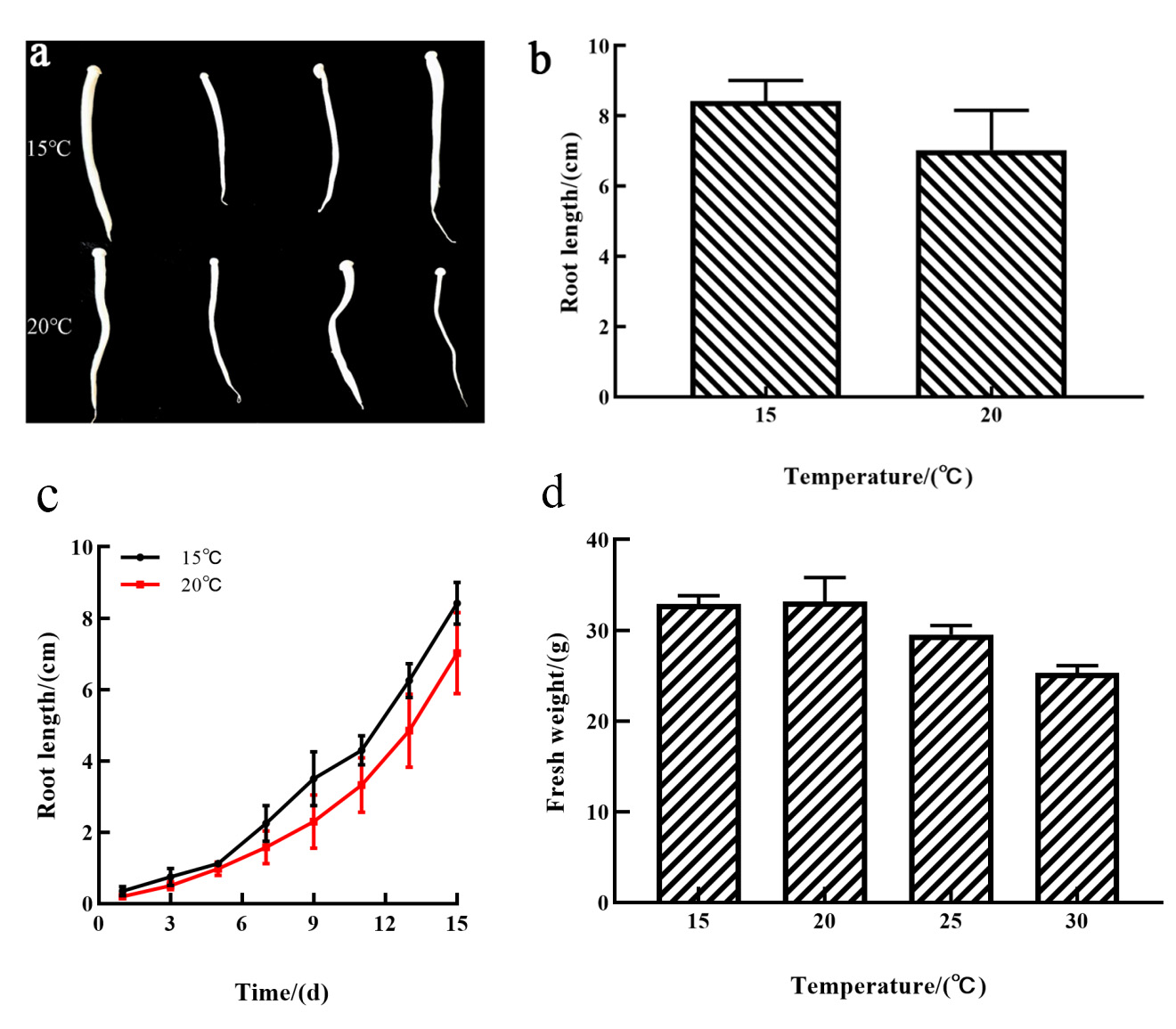
**

***Fig. 1.*** Effects of different temperatures on root length **(a, b)**, growth rate **(c)** and fresh weight **(d)** of the fruiting body

**
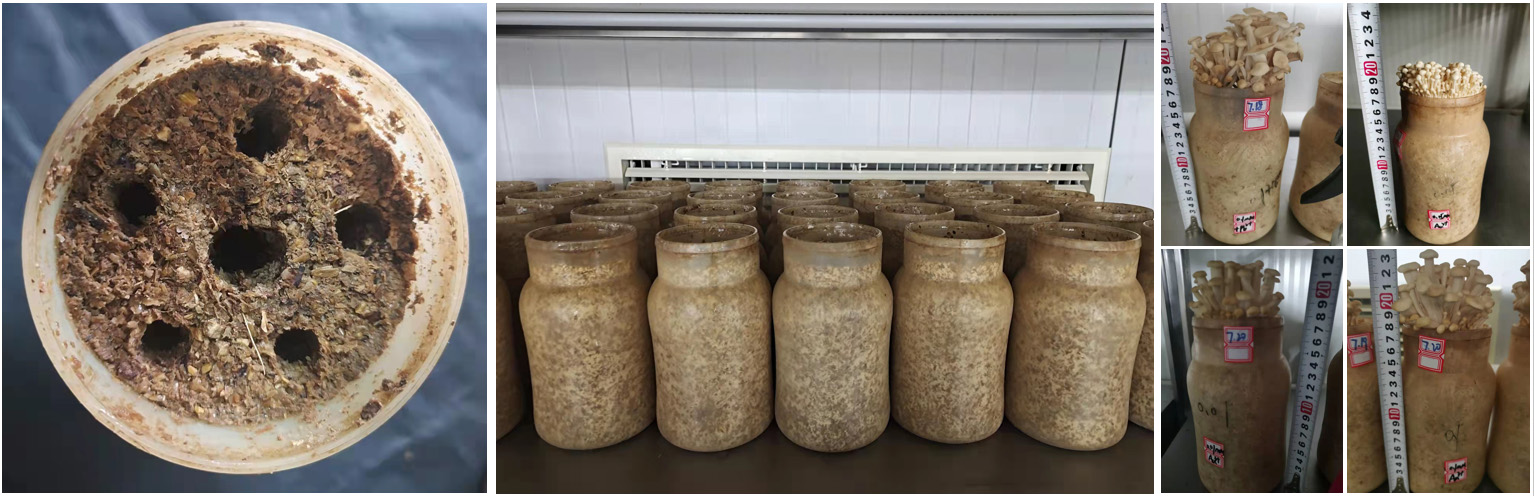
**

***Fig.2.*** Cultivation process of *Flammulina velutipes* fruiting bodies. From left to right, the bottles were filled with nutrients and then holes were punched. Place in the culture chamber for cultivation. Growing enoki mushrooms by spraying with the treatment solution.

**Optimization of HAuCl_4_ concentration**


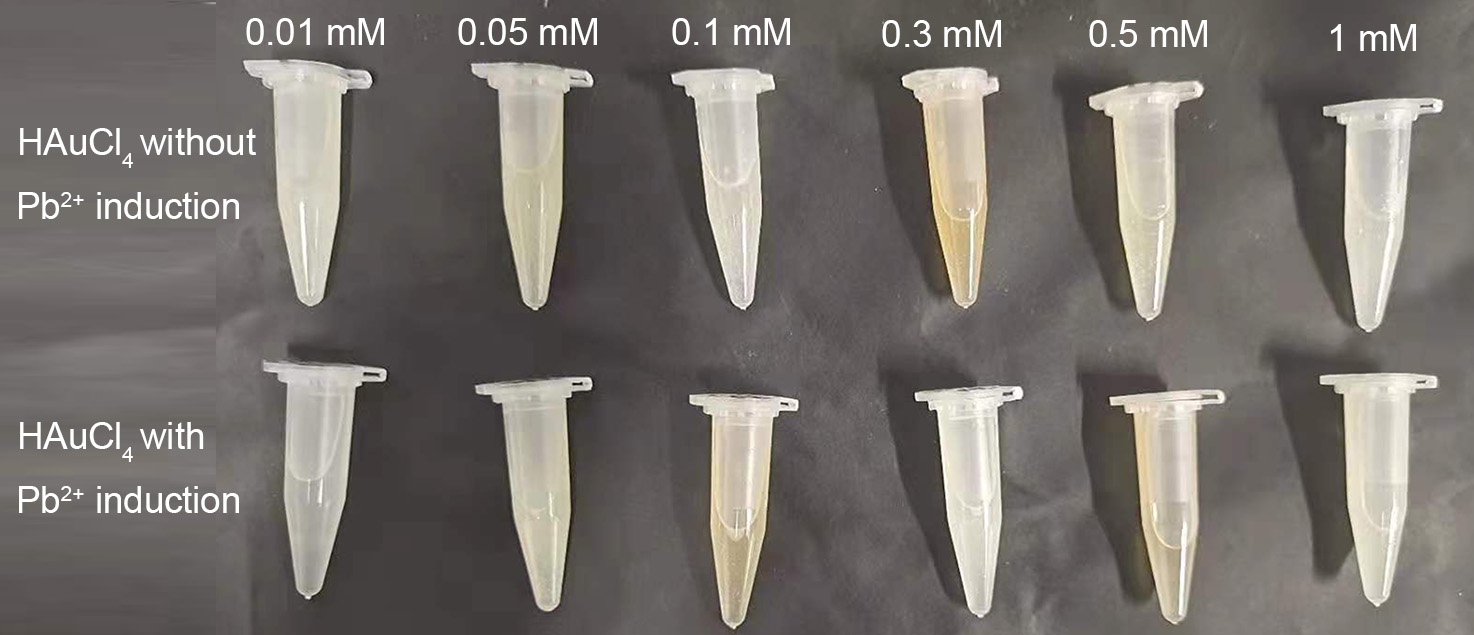


***Fig.3.*** Colour of the enoki mushrooms extracts with HAuCl_4_, with or without Pb^2+^ induction


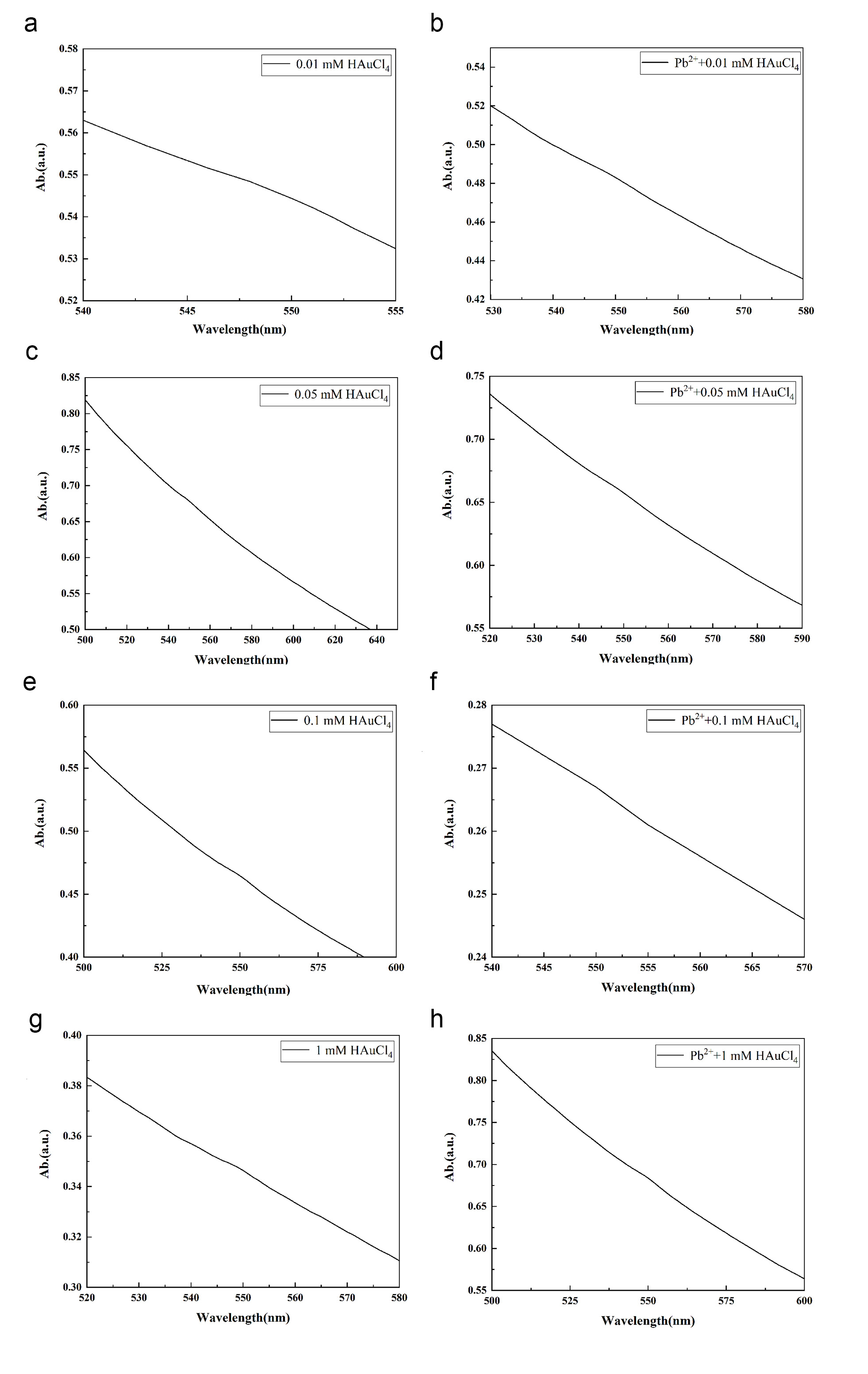


***Fig.4.*** UV-vis absorption spectra of the enoki mushrooms extracts with HAuCl_4_, with or without Pb^2+^ induction
